# Genetic risk score and risk of stage 3 chronic kidney disease

**DOI:** 10.1186/s12882-017-0439-3

**Published:** 2017-01-19

**Authors:** Jiantao Ma, Qiong Yang, Shih-Jen Hwang, Caroline S. Fox, Audrey Y. Chu

**Affiliations:** 1 Framingham Heart Study, Framingham, MA USA; 2 Population Sciences Branch, Division of Intramural Research, National Heart, Lung, and Blood Institute, National Institutes of Health, Bethesda, MD USA; 3 Department of Biostatistics, Boston University, Boston, MA USA

**Keywords:** Genetic risk score, Estimated glomerular filtration rate, Chronic kidney disease

## Abstract

**Background:**

We developed a genetic risk score (GRS) and examined whether the GRS may predict incident stage 3 chronic kidney disease (CKD) independent of common clinical risk factors.

**Method:**

The present study included 2,698 individuals who attended the 15th (1977 to 1979) and the 24th exams (1995 to 1998) in the Framingham Original cohort or the 6th (1995 to 1998) and the 8th exams (2005 to 2008) in the Framingham Offspring cohort. A weighted GRS was constructed combining 53 single nucleotide polymorphisms (SNPs) associated with lower creatinine-based estimated glomerular filtration rate (eGFR). Stage 3 CKD was defined as eGFR <60 mL/min/1.73 m^2^, and incident cases were identified at follow-up after excluding prevalent cases at baseline.

**Results:**

A total of 292 incident cases and 2,406 non-cases were identified over, on average, 11 years of follow-up. After adjustment for sex, age, cohort, baseline eGFR, hypertension, diabetes, and dipstick proteinuria, the odds ratio of incident stage 3 CKD was 1.37 (95%CI: 1.02–1.83) per 10 alleles of the GRS (*P* = 0.04). There was no statistically significant difference between the C-statistic without and with inclusion of the GRS (0.783 and 0.785, respectively; *P* = 0.39).

**Conclusions:**

A GRS developed based on 53 SNPs associated with reduced eGFR was prospectively associated with incident stage 3 CKD. However, this score did not substantially improve discrimination of stage 3 CKD beyond the common clinical risk factors.

**Electronic supplementary material:**

The online version of this article (doi:10.1186/s12882-017-0439-3) contains supplementary material, which is available to authorized users.

## Background

Chronic kidney disease (CKD) contributes to an increasing proportion of public health burden in the US and other parts of the world [1, 2]. Within the US, the prevalence of CKD has steadily increased in the past decades and 13% of American adults are currently affected by CKD [3]. The high prevalence of CKD risk factors such as obesity and type 2 diabetes have contributed to the growing prevalence of CKD [3, 4]. In addition, CKD is associated with elevated risk of cardiovascular disease [5], kidney failure [6], and all-cause mortality [7].

Early detection may help to promote clinical treatment and potentially slow the progression of CKD [8]. However, awareness of CKD is relatively low because individuals may experience no symptoms in the early stages of the disease [9]. Knowledge of common clinical risk factors such as hypertension and diabetes may facilitate identification of individuals at-risk of CKD [4]. In addition to traditional CKD risk factors, there is strong evidence of genetics influencing CKD risk with heritability (*h* ^*2*^) estimates of creatinine-based eGFR of 0.33 in the community-based Framingham Heart Study [10]. Knowledge of a patient’s genetic susceptibility to CKD could potentially enhance the detection of at-risk individuals.

In our prior work, a genetic risk score (GRS), based on 16 single nucleotide polymorphisms (SNPs) previously identified in association with creatinine-based estimated glomerular filtration rate (eGFR) [11], did not improve disease discrimination (C-statistic) for stage 3 CKD beyond common clinical risk factors [12]. We hypothesized that a potential limitation of our prior work was the limited number of loci included in the GRS. Fortunately, recent genome-wide association studies have identified an additional 37 novel loci associated with eGFR [13]. In this study, we created a GRS from this list of 53 SNPs associated with creatinine-based eGFR (16 known and 37 novel SNPs) and examined whether, independent of common clinical risk factors, the updated GRS may predict an increased risk of incident stage 3 CKD in the Framingham Heart Study.

## Methods

### Study participants

The study sample was derived from the Framingham Heart Study’s Original and Offspring cohorts, which have been described previously [14]. The Original cohort recruited 5,209 participants and the Offspring cohort recruited 5,124 participants when the cohorts were initiated. Participants in the two cohorts were evaluated approximately every 3 or 4 years. A total of 8,481 participants had genetic data in the two cohorts. Among these participants, 600 who attended both the 15^th^ (1977–1979) and the 24^th^ (1995–1998) exams of the Original cohort and 2,557 who attended the both the 6^th^ (1995–1998) and the 8^th^ (2005–2008) exams of the Offspring cohort had data available for serum creatinine at baseline or follow-up examinations. We excluded participants who were missing serum creatinine measurements at either exam (*n* = 223) and who had baseline eGFR <60 mL/min/1.73 m^2^ (*n* = 236) for a total of 2,698 participants in the analytic sample. The Framingham Heart Study protocols and procedures were approved by the Institutional Review Board for Human Research at Boston University Medical Center and all participants provided written informed consent.

### CKD status

Stage 3 CKD was defined based on the updated guideline developed by the Kidney Disease Outcomes Quality Initiative, i.e., eGFR <60 mL/min/1.73 m^2^ [15, 16]. Briefly, fasting serum creatinine concentration was measured using either the autoanalyzer technique [17, 18] or the creatinine imodohydrolase assay [19]. In order to reduce potential variability between laboratories, serum creatinine concentration was calibrated using a 2-step process as previously described [4]. The CKD-EPI (Chronic Kidney Disease Epidemiology Collaboration) equation was used to calculate eGFR [20]. Incident stage 3 CKD was defined as new cases identified in the follow-up exams (the 24^th^ exam in the Original cohort and 8^th^ exam in the Offspring cohort) among participants free of stage 3 CKD at baseline (the 15^th^ exam in the Original cohort and 6^th^ exam in the Offspring cohort).

### SNP selection and genotype determination

For the present study, we selected 53 SNPs (Additional file 1: Table S1) that were associated with lower creatinine-based eGFR from a consortium composed of cohorts with European ancestry in the recent CKDGen genome-wide association study [13]. Genotyping was performed with Affymetrix 500 K mapping array and the Affymetrix 50 K supplemental array. Replicated quality control samples yielded high concordance (>99%), with the overall call rate >95%. All variants were imputed to 1000G phase 1 version 3 (2012) using MACH. Genotypes were represented as continuous dosages from 0 to 2. A weighted GRS was developed by summing together the product of the number of risk alleles (eGFR lowering alleles) and the corresponding regression coefficient derived from the CKDGen meta-analysis [13]. For a more intuitive unit of interpretation, the GRS was divided by the sum of regression coefficients (∑_*i* = 1_^*S*3^ *b* = 0.399) and multiplied by the total number of loci (*N* = 53). Therefore, one point of the GRS approximately represented one risk allele and a greater GRS represents higher genetic susceptibility to low eGFR.

### Covariates assessment

At each visit, cardiovascular disease risk factors were measured by following standard protocols [21]. Systolic blood pressure (SBP) and diastolic blood pressure (DBP) were measured twice by same physician, and the average of the two measurements was used. Hypertension was defined as SBP ≥140 mmHg, DBP ≥90 mmHg, or use of anti-hypertensive medication. Type 2 diabetes was defined as fasting plasma glucose ≥126 mg/dl or use of anti-hyperglycemic treatment. A urine dipstick test on a spot urine sample was used to measure proteinuria [22], which was defined when a trace protein or higher was detected.

### Statistical analysis

Participant characteristics were presented as mean ± standard deviation for continuous variables and proportion and counts for dichotomous variables. The prospective association of the GRS and incident stage 3 CKD was analyzed using multiple logistic regression models with generalized estimating equations. The relatedness in our study sample was accounted for using generalized estimating equations. Odds ratio (OR) of incident stage 3 CKD was presented based on increment of 10 alleles of the GRS. Two models were considered: model 1 was adjusted for age, sex, and an indicator of cohort status (Original or Offspring cohort), and model 2 was adjusted for model 1 covariates and CKD risk factors including baseline eGFR, hypertension, type 2 diabetes, and proteinuria. To assess the discrimination capability of the GRS, we performed receiver-operating characteristic curve (ROC) analysis and used a non-parametric model to compare the area under the curve (AUC or the C-statistic) between the fully adjusted model with and without the GRS [23].

In a secondary analysis, we performed the GRS and incident stage 3 CKD analysis stratified by age (<60 and ≥60 years) to assess if the prediction of the GRS is improved among younger as compared to older participants. Additionally, a GRS-by-age (<60 and ≥60 years) interaction term was added to the regression model to assess any potential effect modification. We also categorized GRS into quartiles to investigate the possibility of a threshold effect that may affect the score’s discrimination capability.

All statistical analyses were conducted using R (version 2.13.0; R Foundation) and SAS statistical software (version 9.3; SAS Institute). A two-tailed *P* < 0.05 was considered statistically significant, unless otherwise specified.

## Results

**Fig. 1:**
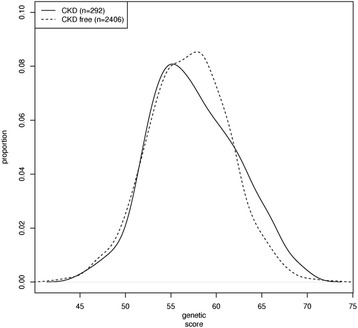
Genetic risk score (53 SNPs) distribution in participants with and without stage 3 chronic kidney disease (CKD). Sample size was 292 for stage 3 CKD cases and 2406 for non-stage 3 CKD cases

Baseline characteristics of the 2,698 participants are presented in Table 1. The mean follow-up period was 11 years. We identified 292 incident stage 3 CKD cases (10.8%) at the follow-up period. The distribution of GRS among incident stage 3 CKD cases and non-cases is shown in Fig. 1. The GRS was slightly higher in incident stage 3 CKD cases compared with non-cases, i.e., those with stage 3 CKD were slightly more genetically predisposed to lower eGFR. The mean GRS was 56.2 ± 4.6 in stage 3 CKD cases and 55.7 ± 4.4 in non-cases (P_difference_ = 0.06).

**Table 1:** Baseline characteristics of participants

	*N* = 2,698
Genetic risk score (106 alleles)	55.8 ± 4.0
Age, years	57.6 ± 8.6
Women, % (*n*)	54.1 (1459)
BMI, kg/m^2^	27.5 ± 4.9
eGFR, mL/min/1.73 m^2^	92.3 ± 24.3
Hypertension, % (*n*)	35.2 (948)
Type 2 diabetes, % (*n*)	6.6 (179)
Dipstick proteinuria, % (*n*)	15.8 (424)

Mean ± standard deviation or proportion (counts)

*eGFR* estimated glomerular filtration rate

**Fig. 2:**
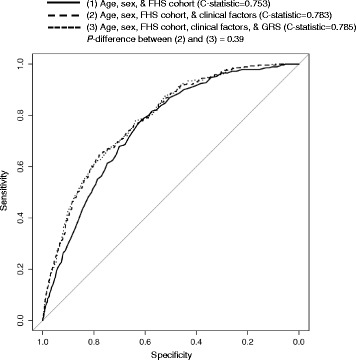
Receiver-operating characteristic curves for stage 3 chronic kidney disease. The C-statistics are based on logistic regression models with adjustment for age, sex, Framingham cohort, and clinical risk factors including baseline estimated glomerular filtration rate, hypertension, type 2 diabetes, and dipstick proteinuria. GRS: genetic risk score

As shown in Table 2, after adjustment for age, sex, and cohort, the odds ratio (OR) of incident stage 3 CKD was 1.44 (95%CI: 1.08, 1.93) per 10 alleles of the GRS, i.e., an average of 44% increase in risk of incident stage 3 CKD, *P* = 0.01. Additional adjustment for baseline clinical CKD risk factors did not substantially attenuate the association between the GRS and incident stage 3 CKD (OR = 1.37, 95%CI: 1.02, 1.83, *P* = 0.04). While the updated GRS did show improved prediction for incident CKD independent of traditional risk factors, we did not observe improved discrimination of the updated GRS in ROC analysis (Fig. 2). In sex-, age-, and cohort-adjusted model, the C-statistic was 0.753, which was increased to 0.783 after additional consideration of baseline eGFR, hypertension, diabetes, and proteinuria, P_difference_ < 0.001. The C-statistic was mostly unchanged in the fully adjusted model without the GRS and with the GRS, 0.783 vs. 0.785, P_difference_ = 0.39.

**Table 2:** Odds ratio (OR) of stage 3 CKD

	Effect size of the genotype score Odds Ratio (95% CI)	*P*-value
Model 1	1.44 (95% CI: 1.08, 1.93)	0.01
Model 2	1.37 (95% CI: 1.02, 1.83)	0.04

Odds ratio and 95% CI were calculated based on increase of per 10 alleles of genetic risk score

Model 1 was adjusted for age, sex, and Framingham cohort

Model 2 was adjusted for age, sex, Framingham cohort, baseline estimated glomerular filtration rate, hypertension, diabetes, and proteinuria

*GRS* genetic risk score

**Fig. 3:**
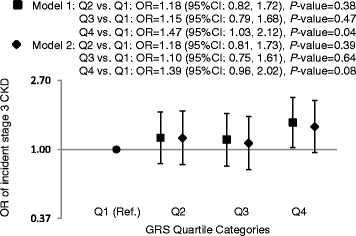
The association of quartile GRS and incident CKD. The GRS ranged from 40.6 to 52.7, 52.8 to 55.8, 55.8 to 58.7, and 55.8 to 71.4 from the lowest to the highest quartile category, respectively. Model 1 was adjusted for age, sex, and Framingham cohort. Model 2 was adjusted for age, sex, Framingham cohort, and clinical risk factors including baseline estimated glomerular filtration rate, hypertension, type 2 diabetes, and dipstick proteinuria. OR: odds ratio; GRS: genetic risk score; CKD: chronic kidney disease. Y axis is in natural logarithmic scale

In secondary analyses, ORs of incident stage 3 CKD using GRS quartiles are presented in Fig. 3. In model 1 (sex- and age-adjusted model), participants in the highest quartile of GRS were at 47% (OR = 1.47, 95% CI: 1.03, 2.12) greater risk of stage 3 CKD compared to those in the lowest quartile of GRS, *P* = 0.04. After additional adjustment for clinical risk factors, OR of incident stage 3 CKD in the top GRS quartile category was 1.39 (95%CI: 0.96, 2.02, *P* = 0.08) compared with the lowest GRS quartile category.

To assess the potential improvement in GRS prediction among the younger age group, we compared the association of the GRS with incident CKD between participants <60 and ≥60 years old. There were 82 incident stage 3 CKD cases and 1,585 non-cases among younger participants, and 210 incident cases and 821 non-cases among older participants. No significant interaction was observed between the GRS and age, P-interaction = 0.89. The ORs of stage 3 CKD in both age groups were similar as that observed in entire study sample, OR = 1.29 (95%CI: 0.75, 2.21, *P* = 0.4) in younger participants and OR = 1.38 (95%CI: 0.96, 1.98, *P* = 0.08) in older participants.

## Discussion

An updated genetic risk score using 53 genetic loci associated with reduced kidney function predicted incident stage 3 CKD in a cohort of middle-aged and older adults with a mean of 11 years of follow-up. Each increment of 10 alleles was associated with a 37% increased risk of CKD adjusted for age, sex and common clinical risk factors. Despite this, the updated GRS did not substantially improve the discrimination of CKD beyond the known clinical risk factors.

In our prior work, we showed that developing a GRS based on 16 genetic loci that were associated with reduced kidney function was not associated with incident CKD after adjustment for clinical risk factors [12]. The present study updated the GRS by adding 37 newly identified genetic loci. We demonstrated that this new GRS constructed from a more comprehensive set of genetic markers was associated with incident CKD after consideration of common clinical risk factors. However, as we have demonstrated before [12], the GRS remained a poor tool for discrimination of incident CKD beyond common clinical risk factors. Similar findings have been observed in many other studies for different phenotypes [24, 25]. In an earlier study, a GRS was constructed using 18 SNPs associated with type 2 diabetes available at that time to predict incident type 2 diabetes cases [26]. However, this study showed the GRS did not much improve the C-statistic beyond simple clinical risk factors. In later studies, more SNPs were included to update the prior GRSs [24, 27, 28], with the latest GRS was composed of 65 type 2 diabetes associated SNPs [28]. However, in all of these later studies, the increase in discrimination was marginal after combining the GRS with common clinical risk factors [24, 27, 28]. Therefore, As suggested by us [12] and many others [24, 27, 28], GRSs developed using common genetic variants that nominally contribute to phenotypic variation may not improve disease discrimination beyond common clinical risk factors alone.

Despite the consistency between our observations and many other studies, the discriminatory power of the newly constructed GRS in the present study may be limited by several aspects. The 37 newly discovered loci have relatively small effect sizes, and the 53 SNPs only explain a small proportion of the variability (3.2%) in eGFR (13). The GRS developed in the present study utilized common genetic variants, i.e., 53 SNPs with minor allele frequency of 0.05 or greater. It is possible that less common or rare SNPs may be discovered from sequencing technologies, and these rare SNPs may represent greater variability in eGFR [29, 30]. However, since they will be rarer, the impact of low frequency variants on overall disease variation in the general population may be similarly low. Incorporating markers from emerging fields such as epigenomics, transcriptomics, or metabolomics may help to develop a multi-disciplinary “omics” risk score that may have sufficient discriminatory power for complex diseases such as CKD. Further investigation into the genetic architecture of renal function and disease may lead to development of a GRS with improved discriminatory capacity.

The present study suggests that common clinical risk factors rather than genetic markers are more useful in disease prediction with our current knowledge. However, we have demonstrated that the GRS is a strong risk factor for stage 3 CKD, independent of clinical risk factors. Additionally, continuing research into the genetic architecture of renal function would provide additional insight into the pathophysiological pathways underlying the development of CKD.

The present data utilized comprehensive clinical data collected from the Framingham Heart Study Original and Offspring cohorts. We were able to examine stage 3 CKD status across 11 years, on average, of follow-up. Some limitations regarding the genetic risk score have been discussed above, e.g., SNPs included in the GRS only accounted for a modest proportion of eGFR heritability. More genetic markers may be discovered in the future, and incorporation of these markers such as rare genetic variants influencing risk of stage 3 CKD may improve the discriminatory ability of the GRS. In addition, our study participants are primarily of European ancestry and therefore the GRS may not be generalizable to other ancestral populations with different allele frequencies and linkage disequilibrium structure.

## Conclusions

Our findings demonstrate that a GRS constructed based on 53 risk alleles for reduced kidney function was associated with incident cases of stage 3 CKD in our adult study sample, however, did not substantially improve disease discrimination beyond clinical risk factors alone. These results emphasize early identification of adverse clinical risk factors should remain a priority for risk prediction in kidney disease.

## Additional file

Additional file 1: Characteristics of SNPs included in the genetic risk score. The supplemental table provides characteristics of the 53 SNPs included in the genetic risk score. (DOC 79 kb)

## Abbreviations

CKD: Chronic kidney disease
eGFR: Estimated glomerular filtration rate
GRS: Genetic risk score
SNP: Single nucleotide polymorphism
