# Supplementary material for: Genetic risk score and risk of stage 3 chronic kidney disease
Source: BMC Nephrol. 2017 Jan 19;18:32. doi: 10.1186/s12882-017-0439-3 (PMC5248454; doi:10.1186/s12882-017-0439-3)
Supplement: Additional file 1: — Characteristics of SNPs included in the genetic risk score. The supplemental table provides characteristics of the 53 SNPs included in the genetic risk score. (DOC 79 kb) [file 12882_2017_439_MOESM1_ESM.doc]

| Supplemental Table. Characteristics of SNPs included in the genetic risk score | | | | | | |
| --- | --- | --- | --- | --- | --- | --- |
| SNP | Chr. | Locus Name | Coded  Allele | Non-coded Allele | OR (95%CI)  Stage 3 CKD | P-value |
| rs1800615 | 1 | *CASP9* | T | C | 0.87 (0.71,1.06) | 0.2 |
| rs12136063 | 1 | *SYPL2* | A | G | 1.03 (0.83,1.27) | 0.8 |
| rs267734 | 1 | *LASS2* | T | C | 1.19 (0.94,1.51) | 0.2 |
| rs3850625 | 1 | *CACNA1S* | A | G | 0.87 (0.64,1.18) | 0.4 |
| rs2802729 | 1 | *SDCCAG8* | A | C | 1.05 (0.86,1.27) | 0.7 |
| rs807601 | 2 | *DDX1* | T | G | 0.80 (0.66,0.97) | 0.02 |
| rs1260326 | 2 | *GCKR* | T | C | 1.06 (0.88,1.28) | 0.5 |
| rs6546838 | 2 | *ALMS1* | A | G | 1.19 (0.94,1.49) | 0.1 |
| rs4667594 | 2 | *LRP2* | A | T | 1.06 (0.88,1.27) | 0.5 |
| rs7422339 | 2 | *CPS1* | A | C | 0.99 (0.81,1.22) | 0.9 |
| rs2712184 | 2 | *IGFBP5* | A | C | 0.96 (0.79,1.15) | 0.6 |
| rs6795744 | 3 | *WNT7A* | A | G | 0.95 (0.69,1.29) | 0.7 |
| rs2861422 | 3 | *TFDP2* | T | C | 0.99 (0.79,1.24) | 0.9 |
| rs9682041 | 3 | *SKIL* | T | C | 0.98 (0.76,1.27) | 0.9 |
| rs10513801 | 3 | *ETV5* | T | G | 0.99 (0.73,1.34) | 0.9 |
| rs17319721 | 4 | *SHROOM3* | A | G | 0.94 (0.78,1.12) | 0.5 |
| rs228611 | 4 | *NFKB1* | A | G | 0.97 (0.80,1.18) | 0.8 |
| rs11959928 | 5 | *DAB2* | A | T | 0.88 (0.72,1.07) | 0.2 |
| rs6420094 | 5 | *SLC34A1* | A | G | 1.08 (0.77,1.52) | 0.6 |
| rs7759001 | 6 | *ZNF204* | A | G | 0.89 (0.72,1.10) | 0.3 |
| rs9472135 | 6 | *VEGFA* | T | C | 0.96 (0.77,1.19) | 0.7 |
| rs316009 | 6 | *SLC22A2* | T | C | 0.80 (0.57,1.12) | 0.2 |
| rs10277115 | 7 | *UNCX* | A | T | 1.28 (0.84,1.97) | 0.3 |
| rs3750082 | 7 | *KBTBD2* | A | T | 0.97 (0.79,1.18) | 0.8 |
| rs848490 | 7 | *TMEM60* | C | G | 1.14 (0.91,1.42) | 0.2 |
| rs7805747 | 7 | *PRKAG2* | A | G | 1.31 (1.08,1.58) | 0.01 |
| rs6459680 | 7 | *RNF32* | T | G | 0.91 (0.74,1.13) | 0.4 |
| rs3758086 | 8 | *STC1* | A | G | 1.24 (1.03,1.50) | 0.02 |
| rs4744712 | 9 | *PIP5K1B* | A | C | 0.92 (0.76,1.11) | 0.4 |
| rs1044261 | 10 | *WDR37* | T | C | 1.03 (0.72,1.46) | 0.9 |
| rs10994860 | 10 | *A1CF* | T | C | 0.87 (0.67,1.13) | 0.3 |
| rs163160 | 11 | *KCNQ1* | A | G | 0.97 (0.76,1.25) | 0.8 |
| rs963837 | 11 | *MPPED2* | T | C | 1.11 (0.91,1.36) | 0.3 |
| rs4014195 | 11 | *AP5B1* | C | G | 1.13 (0.91,1.40) | 0.3 |
| rs10774021 | 12 | *SLC6A13* | T | C | 0.97 (0.80,1.17) | 0.7 |
| rs10491967 | 12 | *TSPAN9* | A | G | 1.06 (0.75,1.49) | 0.7 |
| rs7956634 | 12 | *PTPRO* | T | C | 1.11 (0.86,1.43) | 0.4 |
| rs1106766 | 12 | *INHBC* | T | C | 1.09 (0.88,1.35) | 0.4 |
| rs716877 | 13 | *DACH1* | C | G | 1.07 (0.87,1.32) | 0.5 |
| rs476633 | 15 | *INO80* | C | G | 0.95 (0.78,1.16) | 0.6 |
| rs2467853 | 15 | *GATM* | T | G | 1.01 (0.83,1.24) | 0.9 |
| rs491567 | 15 | *WDR72* | A | C | 1.10 (0.88,1.38) | 0.4 |
| rs1394125 | 15 | *UBE2Q2* | A | G | 1.15 (0.86,1.52) | 0.4 |
| rs13329952 | 16 | *UMOD* | T | C | 1.28 (1.01,1.63) | 0.04 |
| rs164748 | 16 | *DPEP1* | C | G | 0.92 (0.77,1.10) | 0.4 |
| rs2453580 | 17 | *SLC47A1* | T | C | 1.03 (0.85,1.25) | 0.7 |
| Supplemental Table 1. Continued | | | | | | |
| rs9916302 | 17 | *CDK12/FBXL20* | T | C | 1.25 (0.99,1.58) | 0.06 |
| rs11657044 | 17 | *BCAS3* | T | C | 1.12 (0.85,1.48) | 0.4 |
| rs8091180 | 18 | *NFATC1* | A | G | 0.67 (0.27,1.67) | 0.4 |
| rs12460876 | 19 | *SLC7A9* | T | C | 1.17 (0.97,1.42) | 0.1 |
| rs11666497 | 19 | *SIPA1L3* | T | C | 0.96 (0.77,1.21) | 0.7 |
| rs6088580 | 20 | *TP53INP2* | C | G | 1.12 (0.93,1.35) | 0.2 |
| rs17216707 | 20 | *BCAS1* | T | C | 0.83 (0.65,1.06) | 0.1 |
| Odds ratio (95% confidence interval) and P-values were calculated from prospective association in the Framingham Heart Study. | | | | | | |
